# Segmentation of tobacco shred point cloud and 3-D measurement based on improved PointNet++ network with DTC algorithm

**DOI:** 10.3389/fpls.2024.1508449

**Published:** 2025-01-21

**Authors:** Yihang Wang, Haiwei Zheng, Jie Yang, Yan Wang, Li Wang, Qunfeng Niu

**Affiliations:** ^1^ School of Electrical Engineering, Henan University of Technology, Zhengzhou, China; ^2^ Guangxi China Tobacco Industry Co., Ltd, Cigarette Process Quality Department, Guangxi, China; ^3^ Henan Centerline Electronic Technology Co., Ltd, R&D Department, Zhengzhou, China; ^4^ Institute for Complexity Science, Henan University of Technology, Zhengzhou, China

**Keywords:** blended tobacco shred, PointNet++, semantic segmentation, non-contact measurement, DTC

## Abstract

**Introduction:**

The three dimensions of the tobacco silk components (cut stem, tobacco silk, reconstituted tobacco shred, and expanded tobacco silk) of cigarettes directly affect cigarette combustibility; by accurately measuring the dimensions of different tobacco silks in cigarettes, it is possible to optimize combustibility and reduce the production of harmful substances. Identifying the components of tobacco shred in cigarettes is a prerequisite for three-dimensional measurement. The two-dimensional image method can identify the tobacco shred and measure its two-dimensional characteristics but cannot determine its thickness. This study therefore focuses on the identification of the tobacco shred and measuring it in three dimensions.

**Methods:**

The point cloud data of the upper and lower surfaces of tobacco shred are segmented using the improved three-dimensional point cloud segmentation model based on the PointNet++ network. This model combines the weighted cross-entropy loss function to enhance the classification effect, the cosine annealing algorithm to optimize the training process, and the improved k-nearest neighbors multi-scale grouping method to enhance the model’s ability to segment the point cloud with complex morphology. Meanwhile, this study also proposes a dimension transformation calculation method for calculating the three dimensions of tobacco shred.

**Results:**

The experimental results show that the precision and recall of the improved segmentation model increased from 84.27% and 83.63% to 95.13% and 97.68%, respectively; the relative errors of the length and width of tobacco shred were less than 5% and 7%, and the relative error of the standard gauge block thickness measurement reached 1.12%.

**Discussion:**

This study also provides a new idea for implementing three-dimensional measurements of other flexible materials.

## 1 Introduction

The Framework Convention on Tobacco Control (FCTC) Articles 9 and 10 mandate that signatories regulate the contents and emissions of tobacco products and establish corresponding testing and measurement methods. Manufacturers and importers are required to disclose information about these ingredients and emissions to government authorities and the public (Acuña, 2017). The physical characteristics of tobacco shred components (such as cut stem, tobacco silk, reconstituted tobacco shred, and expanded tobacco silk)—including length, width, and thickness—significantly affect cigarettes’ physical properties, smoke characteristics, and sensory quality (State Tobacco Monopoly Administration, 2009; Wang et al., 2020). Therefore, achieving precise and efficient identification of tobacco shred types and determination of their dimensional measurements is crucial for exploring blend design and evaluating tobacco quality (Qi et al., 2022).

To measure the physical characteristics of mixed tobacco shreds, it is essential first to identify and segment the components of the cigarette’s tobacco shred blend. Zhang (2013) successfully predicted the blend ratio of expanded and sheet tobacco shreds by combining Fourier transform near-infrared spectroscopy with partial least squares regression (PLS). The model achieved a correlation coefficient of 0.993, demonstrating its efficacy and accuracy in rapidly detecting and monitoring the uniformity of expanded tobacco shred blending during the cutting process. Mei et al. (2021) used near-infrared hyperspectral imaging combined with second-derivative and Savitsky–Golay smoothing methods to verify the effectiveness of a component identification model based on both pixel- and sample-based approaches, with an identification rate of 86.97%. Research methods for identifying tobacco shred components primarily utilize terahertz time-domain spectroscopy, near-infrared hyperspectral imaging, and machine vision technologies. Zhou et al. (2022) used terahertz time-domain spectroscopy combined with low-variance filtering, principal component analysis (PCA) feature extraction, and classification models to successfully discriminate three tobacco blend components: tobacco silk, cut stem, and reconstituted tobacco shred.

In recent years, machine vision has rapidly advanced alongside computational technology, with the introduction of deep learning significantly improving image processing efficiency and accuracy. Three critical tasks within this domain include image classification (He et al., 2016; Wu et al., 2019; Sengupta et al., 2019), object detection (Jiang and Learned-Miller, 2017; Redmon, 2016; Li et al., 2020), and image segmentation (Lu et al., 2019; Williams et al., 2023; Bharati and Pramanik, 2020). Image classification assigns categories, while detection and segmentation identify objects and precisely delineate their boundaries. These technological advances offer robust support for a wide range of applications.

The combination of machine vision and deep learning has also been extensively studied with respect to identifying and segmenting tobacco shred types. To enhance sorting efficiency and classification effectiveness, Niu et al. (2022a) developed a tobacco shred recognition method that combines machine vision and deep learning. The method achieved a 95.5% accuracy rate using an improved Light-VGG network on a custom-built database of four tobacco shred categories, significantly reducing model parameters and prediction time compared to VGG16. Liu et al. (2022) proposed a tobacco shred recognition method based on an efficient channel attention mechanism and multi-scale feature fusion, achieving 97.23% accuracy and a 0.107-s per-image detection time with the improved Inception-ResNet-V2 network.

Niu et al. (2022b) introduced a novel tobacco shred classification method based on the MS-X-ResNet network, achieving 96.56% accuracy at 103 milliseconds per image, offering a new solution for real-time identification in tobacco production quality control. Wang et al. (2023) proposed a tobacco shred image segmentation model based on the improved Mask R-CNN network and applied the chain-of-thought (COT) algorithm to resolve overlap in shred identification and area calculation. It achieved 90.2% detection accuracy and 89.1% segmentation accuracy on overlapping tobacco shred datasets, improving the actual detection rate in overlapping regions from 81.2% to 90%. Jia et al. (2023) developed a new multi-target detection model based on the improved YOLOv7-tiny model to identify and calculate the unbroken ratio of mixed tobacco shreds, achieving a detection accuracy of 0.883 and a testing time of 4.12 milliseconds, providing an efficient method for multi-target detection and dimensional measurement in tobacco quality inspection lines.

Although the above methods have demonstrated effectiveness in recognizing and segmenting tobacco filaments, and studies have been conducted to measure the length and width of tobacco filaments, these efforts have been limited to the realm of 2-D image processing. Given tobacco’s complex bending characteristics, it is difficult to accurately calculate the actual length and width of filaments, let alone measure their thickness, using 2-D images alone. Therefore, the currently available methods have great limitations with respect to accurately measuring all three dimensions of tobacco filaments.

Three-dimensional laser scanning technology can capture detailed 3-D point cloud data to provide a comprehensive representation of an object’s geometry. Based on these data, advanced deep learning networks have been developed specifically for the purpose of point cloud processing to enable accurate recognition and segmentation. These techniques utilize deep learning and segmentation methods based on 3-D point clouds (Lu et al., 2024) to efficiently analyze complex 3-D data and extract rich feature information, providing powerful technical support for accurate measurements. Compared with traditional 2-D image processing, 3-D networks can more accurately capture the geometric shapes and spatial structures of objects, thereby improving the precision of shred segmentation and recognition. However, the sparse, irregular, high-dimensional, large-scale nature of point cloud data, along with noise and outliers, presents challenges in processing and recognizing 3-D data, requiring advanced algorithms and computational resources (Shi et al., 2024).

PointNet is a novel neural network designed to directly process point cloud data, emphasizing the invariance of point order and achieving outstanding results in tasks such as object classification, part segmentation, and scene semantic parsing while providing a theoretical analysis of network learning and robustness (Qi et al., 2017a). PointNet++ builds on this by introducing hierarchical layers that recursively partition and adaptively fuse features, effectively learning point clouds’ local features and significantly outperforming state-of-the-art methods in terms of 3-D point cloud benchmarks (Qi et al., 2017b). PointNeXt was also introduced, incorporating improved training strategies and reverse residual bottleneck designs, achieving superior performance in 3-D classification and segmentation tasks while also being ten times faster in inference (Qian et al., 2022). However, few studies have been conducted on methods to obtain three-dimensional dimensional information on tiny tobacco filaments.

In this study, we chose to use the PointNet++ model for point cloud segmentation because of its ability to handle complex geometric shapes and its efficient multi-scale grouping mechanism, which is essential for capturing both local and global features in point clouds. While other models, such as PointNet and DGCNN (Wang et al., 2019), also offer promising performance, PointNet++’s hierarchical structure and improved feature aggregation techniques make it particularly suitable for accurately segmenting the tobacco shreds in our dataset. Furthermore, PointNet++ is robust in dealing with varying point cloud densities and irregularities, giving it a distinct advantage over voxel-based methods like VoxelNet (Zhou and Tuzel, 2018). To tackle the challenge of accurately measuring tobacco shreds in a mixed environment, this study leveraged 3-D laser scanning technology in conjunction with an improved deep learning model to segment and measure the three dimensions of the shreds.

We propose an improved PointNet++ point cloud segmentation model to efficiently process and analyze the complex 3-D data, extracting rich feature information to enable accurate identification and segmentation. Additionally, a new 3-D measurement method is introduced to precisely measure the length, width, and thickness of the tobacco shreds. Our main contributions are as follows:

- By improving PointNet++’s multi-scale grouping method and integrating k-nearest neighbors (KNN), more stable feature extraction is achieved. In addition, a weighted cross-entropy loss function is introduced to enhance minority class classification precision, and a cosine annealing algorithm is used to optimize the learning rate adjustment strategy, significantly improving classification accuracy, model convergence speed, and overall training results on complex geometric point clouds.
- We constructed a multi-category point cloud dataset encompassing five main categories: Cut Stem, Tobacco Silk, Reconstituted Tobacco Shred, Expanded Tobacco Silk, and Background Noise. Each category had over 1,500 samples, ensuring a robust training set.
- Traditional 2-D image methods struggle to accurately measure the actual dimensions of tobacco shreds due to their softness. We proposed the dimension transformation calculation (DTC) algorithm, which, through dimensional transformation and mapping, accurately calculates the length, width, and thickness of tobacco shreds, significantly improving measurement precision.
- This approach offers a new method for the three-dimensional measurement of materials like tobacco shreds, which are flexible, curved, and require non-contact measurement.

This paper is structured as follows: Section 2 describes the point cloud acquisition device and builds a dataset of tobacco shred components. Section 3 describes the improved PointNet++ network and the DTC algorithm. Section 4 conducts experiments based on the improved PointNet++ network and the DTC algorithm to evaluate the performance of the segmentation model and the accuracy of the measurement algorithm. Section 5 summarizes the work of this paper and provides an outlook.

## 2 Methodology overview

**Figure 1:**
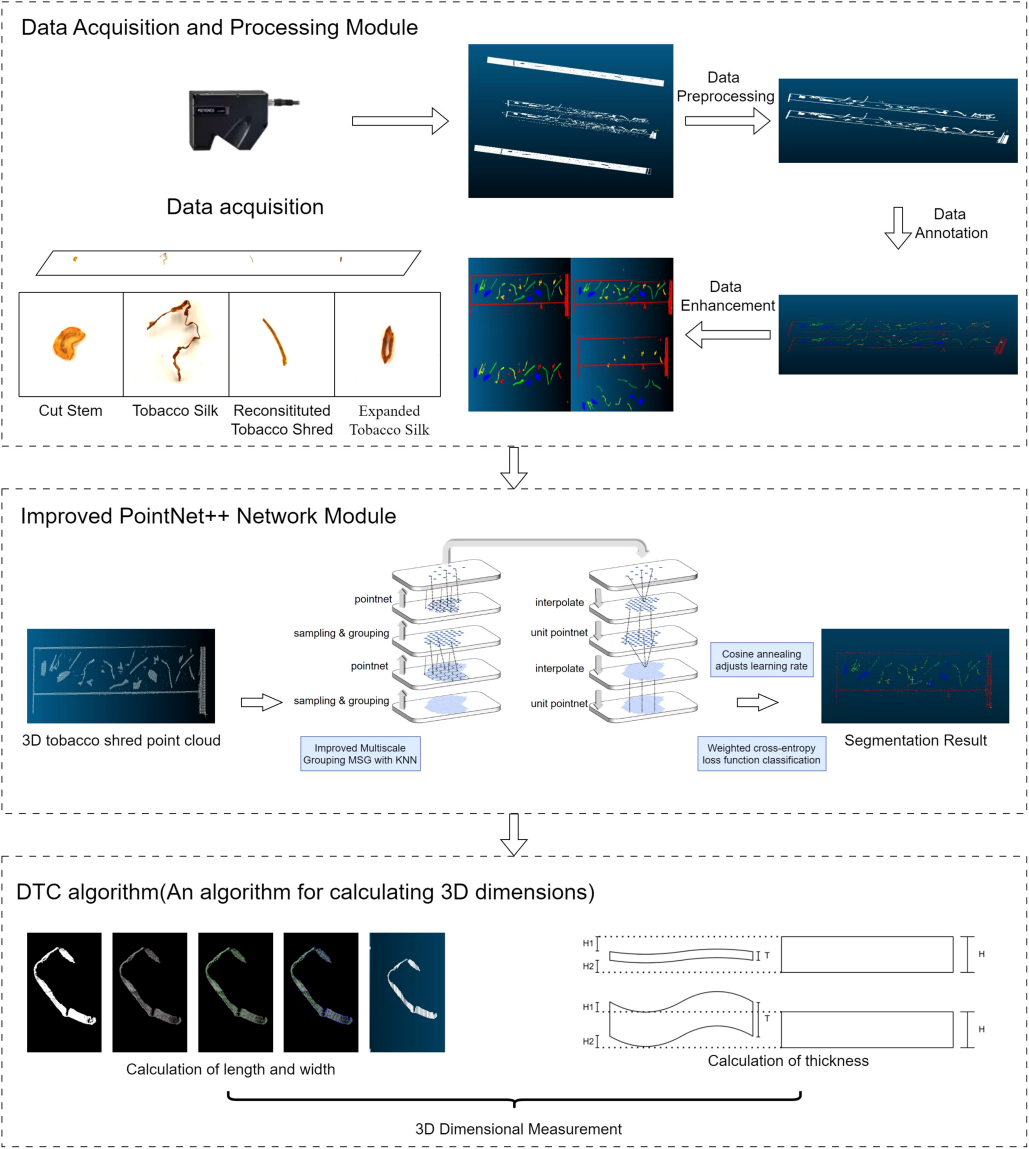
Overall system framework.

Our proposed hybrid tobacco shred part-segmentation and 3-D measurement method includes three main modules: a data acquisition and processing module, a deep learning module, and a 3-D measurement module. The overall framework of the system is shown in **Figure 1**. The data acquisition and processing module utilizes a point cloud data acquisition system for collecting and pre-processing data. To enhance the deep learning model’s performance and generalization ability and reduce the risk of overfitting, annotated data are optimized using data augmentation techniques. The deep learning module is based on an improved PointNet++ network in which the loss function was enhanced and the learning rate dynamically adjusted using the cosine annealing algorithm to improve model accuracy. Additionally, PointNet++’s multi-scale grouping method was optimized for better feature extraction. Finally, the 3-D measurement module calculates the length, width, and thickness of the segmented tobacco shreds using the DTC algorithm.

The tobacco used in this study was provided by Jiangxi China Tobacco Industry Co. The experimental methodology commenced with preprocessing the point cloud data acquired from the tobacco filaments and inputting them into the improved PointNet++ for 3-D point cloud segmentation. The segmented tobacco was classified into different categories and aligned for measurement. The specific experimental process is detailed in **Supplementary Figure 1**.

### 2.1 Data acquisition

**Figure 2:**
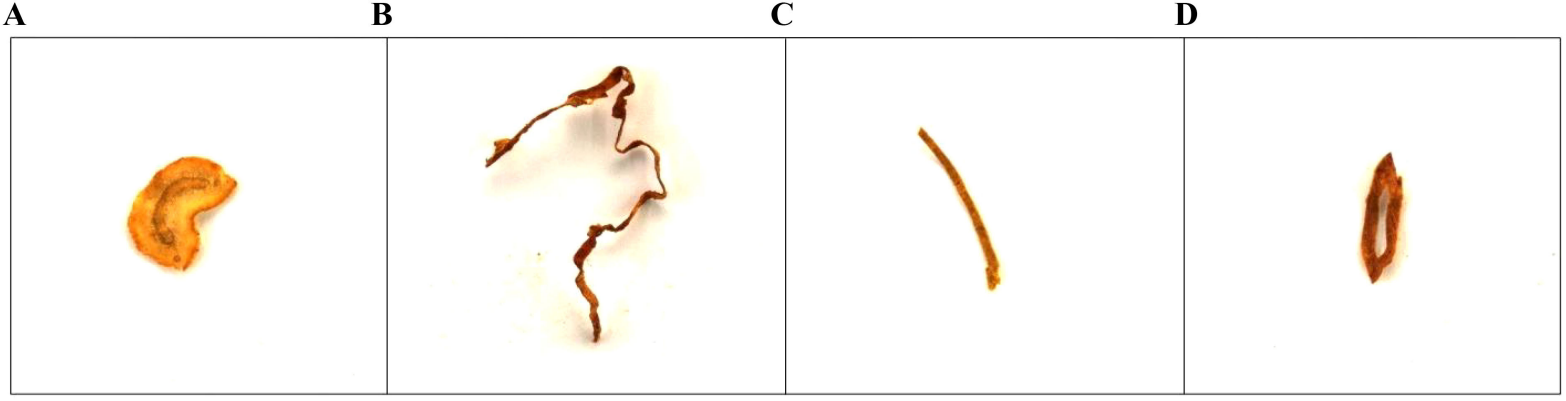
Types of tobacco shreds: **(A)** Cut Stem; **(B)** Tobacco Silk; **(C)** Reconsitituted Tobacco Shred; **(D)** Expanded Tobacco Silk.

The tobacco shred samples used in this study were obtained from the cigarette production line after the silk-making and flavoring processes had taken place and before the cigarettes had been rolled. The four main tobacco shred types in cigarettes are stem shreds, leaf shreds, reconstituted tobacco shreds, and expanded tobacco shreds, as shown in **Figure 2**. Stem shreds are fine strips of tobacco stem with a soft texture and small cutting thickness that are processed to a certain width after cutting. Leaf shreds are made by cutting tobacco leaves and have natural leaf vein textures on the surface, serving as the main component of cigarettes. Reconstituted shreds are made from reprocessing tobacco waste, fragments, and dust into tobacco shreds of a certain shape and specification. Expanded tobacco shreds have been processed to expand the volume of the tobacco leaves by adding expansion agents during tobacco processing.

To acquire high-quality, stable point cloud data of tobacco shreds in 3-D form, a high-precision laser line scanning system with XYZ three-axis displacement was designed. The system consists of a laser sensor, laser controller, XY-axis displacement mechanism, Z-axis rotation mechanism, motion controller, stage, precision ceramic block, and computer. The laser sensor model LJ-X8080 was developed and manufactured by Keyence Corporation, a company headquartered in Japan. This equipment controls the laser emission and data transmission via a laser controller. A 405 nm blue laser is emitted onto the object’s surface; part of the light is reflected by the surface and received by the sensor’s internal chip, which then processes the light signals to calculate the distance between the object’s surface and the sensor. The sensor has a static precision of 0.5 μm in the Z-axis direction and 2.5 μm in the X-axis direction, making it capable of scanning fine textures and other morphological features of tobacco shreds.

The motion controller drives the XY-axis platform to move back and forth and left and right within a certain range to capture scanning data of the tobacco shred surface. The Z-axis rotation mechanism independently controls the laser sensor to rotate around the circumference to scan the tobacco shreds from both top and bottom. The stage consists of high-transparency glass to minimize the impact of light refraction. The overall point cloud data acquisition system is shown in **Supplementary Figure 2**.

### 2.2 Data preprocessing

#### 2.2.1 Point cloud data preprocessing

Point cloud data preprocessing plays a crucial role in deep learning. Raw point cloud data often contain numerous discrete points, isolated points, and irrelevant background information, all of which can negatively impact the training effectiveness and inference speed of deep learning models. Appropriate preprocessing steps are therefore essential for enhancing model performance. This study proposes a point cloud preprocessing method based on spatial coordinate cropping. By utilizing the difference in Z-coordinates between redundant background points and the target tobacco shreds, data outside the Z-coordinate range of the tobacco shreds can be cropped and discarded to define the region of interest (ROI). After extracting the ROI, radius filtering and Gaussian filtering are applied to the point cloud data. The radius filter removes isolated points and noise by evaluating point density within a specified radius and discarding points with fewer neighbors than a set threshold. This process preserves the dense structure of the tobacco shreds while eliminating background noise. The Gaussian filter smooths the data, reducing high-frequency noise while maintaining important features such as edges and surface continuity. Finally, downsampling is applied to significantly reduce the data volume—after preprocessing, the point cloud data volume is only about 9% of the original data size. This preprocessing method greatly enhances the efficiency of subsequent data processing and analysis, enabling deep learning models to process higher-quality data faster. The point cloud preprocessing flow is shown in **Supplementary Figure 3**.

#### 2.2.2 Data labeling

**Figure 3:**
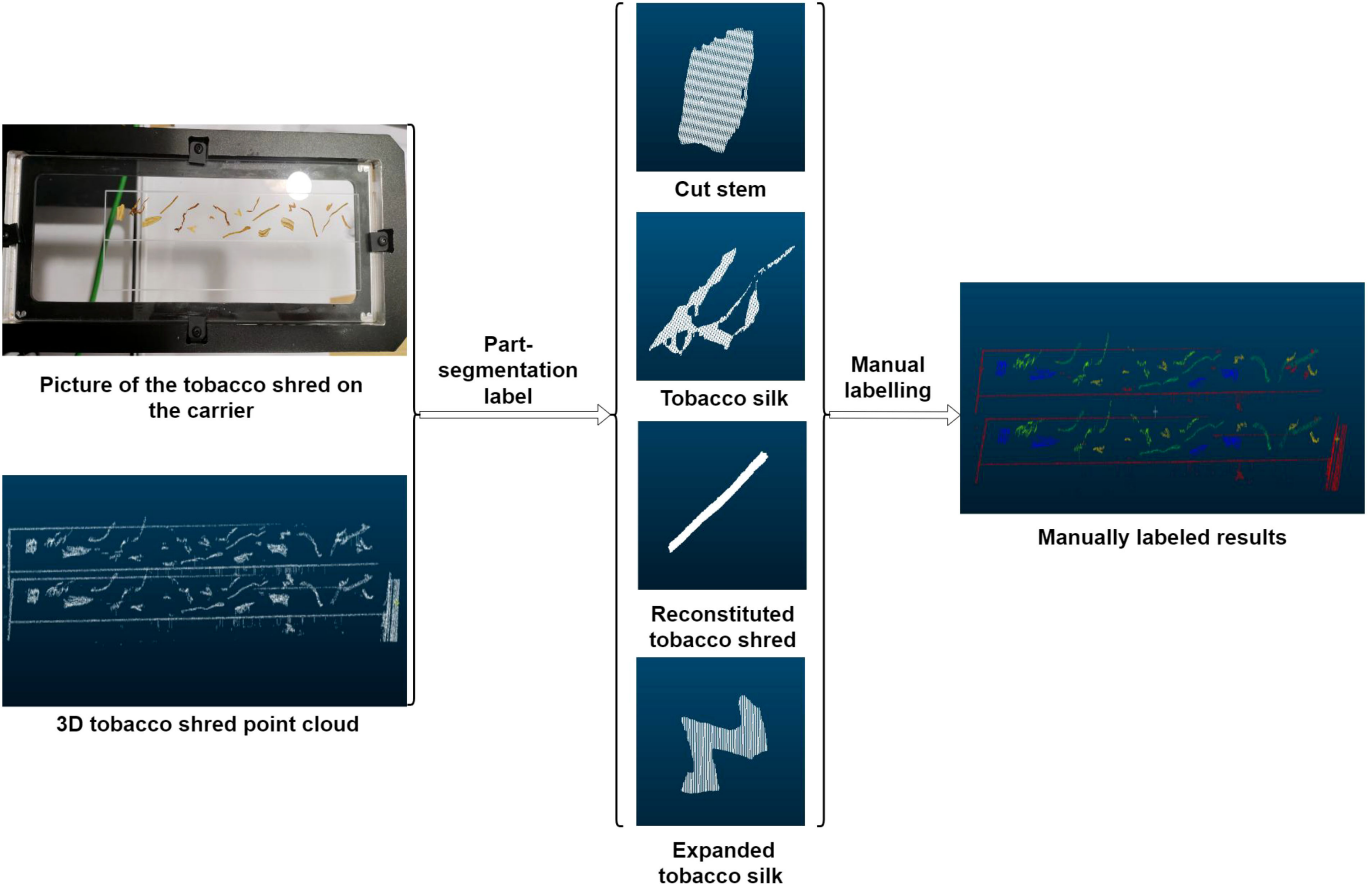
Methods of data labelling.

In deep learning-based point cloud segmentation methods, a certain number of manually labeled samples are required to construct the training dataset. However, because of the challenges of intuitively recognizing point cloud data in 3-D space, direct labeling based solely on point cloud data is prone to errors. To address this issue, it is essential to photograph tobacco samples prior to collecting their corresponding point cloud data. This facilitates comparison during the labeling process, ensuring greater accuracy. The detailed labeling workflow is illustrated in **Figure 3**.

Comparing physical images of tobacco shreds to identify key points, the CloudCompare software was used for data labeling of the shreds. The four types of tobacco shreds were labeled as follows: stem shreds as “0,” reconstituted tobacco shreds as “1,” leaf shreds as “2,” and expanded shreds as “3.” After labeling the target tobacco shreds, the remaining background and unprocessed noise were labeled as “4,” as shown in **Table 1**.

**Table 1:** Tobacco shred dataset labels.

Label	Classes of datasets	Quantity
0	Cut Stem	Over 1500
1	Tobacco Silk	Over 1500
2	Reconsitituted Tobacco Shred	Over 1500
3	Expanded Tobacco Silk	Over 1500
4	Background Noise	Unknown

#### 2.2.3 Data augmentation strategy

Because of the small amount of original point cloud data and its sparse, noisy, and partially missing nature, deep learning models are prone to overfitting during training, leading to poor performance when processing new data. Data augmentation can simulate these situations, generating different forms of point cloud data, which not only improves model accuracy but also helps the model learn how to handle these noises and defects during training, thus improving model robustness and generalization ability.

The point set in the labeled point cloud data is denoted as P, and the expression of any point pi in the point set is ${\left(x,y,z,l\right)}^{T}$ , where x,y,z are the relative coordinates of the point cloud, representing the shape and position information of the object or scene surface. The label number of the point cloud is l, with a value range of 0, 1, 2, 3, 4. The data augmentation process includes operations such as global random displacement, global random rotation, local random rotation, label random rotation, and background label removal on any point pi in the point set P. The augmented data are then analyzed in subsequent experiments.

• Global Random Displacement. Global random displacement adds a random displacement value Δ to the coordinate components (x,y,z) of any point pi in the tobacco shred data point set, denoted as Δx,Δy,Δz . The random displacement value Δ is randomly obtained using Python’s random function, with a value range of [−0.1,0.1] . The value of the label l is then unchanged, so the global random displacement transformation formula is as follows:


(1) $$\begin{array}{c} \left[\begin{array}{c} x_{\Delta }\\ y_{\Delta }\\ \begin{array}{c} z_{\Delta }\\ l_{\Delta } \end{array} \end{array}\right]=\left[\begin{array}{c} x\\ y\\ \begin{array}{c} z\\ l \end{array} \end{array}\right]+\left[\begin{array}{c} \Delta_{x}\\ \Delta_{x}\\ \begin{array}{c} \Delta_{z}\\ 0 \end{array} \end{array}\right]\\ \Delta =rand\left(-0.1,0.1\right) \end{array}$$

• Global Random Rotation. The global random rotation operation rotates the coordinate component (x,y,z) of any point pi in the smoke data point set around the X-axis, Y-axis, or Z-axis by a random angle θ. The value of the random rotation angle θ ranges from [0, 2π]. During the rotation process, although the spatial locations of the points are changed, their associated labeled values do not change with the transformation of the rotation matrix. This means that no matter how the data points are rotated, the smoke feature or category information represented by each point remains the same. The experiments in this paper use rotation around the Y-axis for each point in the point set, so the global random rotation formula is as follows:


(2) $$\begin{array}{c} \left[\begin{array}{c} x_{\theta }\\ y_{\theta }\\ \begin{array}{c} z_{\theta }\\ l_{\theta } \end{array} \end{array}\right]=\left[\begin{array}{ccc} \begin{array}{c} cos\left(\theta \right)\\ 0\\ \begin{array}{c} -sin\left(\theta \right)\\ 0 \end{array} \end{array} & \begin{array}{c} 0\\ 1\\ \begin{array}{c} 0\\ 0 \end{array} \end{array} & \begin{array}{cc} \begin{array}{c} sin\left(\theta \right)\\ 0\\ \begin{array}{c} cos\left(\theta \right)\\ 0 \end{array} \end{array} & \begin{array}{c} 0\\ 0\\ \begin{array}{c} 0\\ 1 \end{array} \end{array} \end{array} \end{array}\right]+\left[\begin{array}{c} \Delta_{x}\\ \Delta_{x}\\ \begin{array}{c} \Delta_{z}\\ 0 \end{array} \end{array}\right]\\ \theta =rand\left(0,2\pi \right) \end{array}$$

• Label Random Rotation. Label random rotation refers to rotating only the tobacco shred data points associated with a specific label value by a random angle while keeping the background area unchanged. This operation aims to simulate or enhance the dataset’s diversity while ensuring the consistency of background information so that the model can accurately recognize and process tobacco shred features during subsequent training or analysis. The formula for this experiment is the same as that for the global random rotation but with a different label selection for rotation.

• Removal of Background and Noise. By data labeling the acquired tobacco shred data, it is easy to observe that label 4, representing background and noise, occupies approximately half of the total data volume. After removing all data with label 4, a clean tobacco shred dataset was obtained. This dataset contains only data points related to tobacco shred features, more accurately reflecting their actual characteristics, reducing interference and misjudgment caused by background and noise, and improving the model’s generalization ability.

## 3 Improved PointNet++ and 3-D size measurement

### 3.1 Improved PointNet++ Algorithm

#### 3.1.1 Optimizing PointNet++ with a weighted cross-entropy loss function

The loss function is used to measure the difference between the model’s predicted results and the actual target values, guiding the optimization of model parameters and thereby improving model prediction accuracy. In the training process, the optimization algorithm adjusts the model parameters to minimize the loss function, making the model’s predictions closer to the actual values. Point cloud segmentation is a multi-class classification problem for each point. Therefore, the cross-entropy loss function is used to train the deep network model. However, experiments have shown that when some classes have much more training data than others, the standard cross-entropy loss function may cause the model to prioritize learning the majority classes, with the minority classes contributing relatively little, making it difficult to effectively adjust the model parameters. In this experiment, the total data volume of tobacco shreds is only half that of the background and noise. Although the overall accuracy of the model may be high, the prediction performance for minority classes is poor. To address this issue, we used a weighted cross-entropy loss function to optimize the model by assigning different weights to the tobacco shreds and backgrounds to improve the classification accuracy. The formula for the weighted cross-entropy loss function is as follows:


(3) $$L_{wce}=-\frac{1}{N}\sum_{i=1}^{N}\sum_{j=0}^{\text{C}}w_{j}y_{i,j}\log p_{i,j}$$

In Equation 3, N denotes the number of samples, C is the number of categories, $W_{j}$ denotes the weight of the jth category, $y_{i,j}$ is an element of the one-hot coding vector—i.e., it denotes the probability (0 or 1) that the true category of the ith sample is j—and $p_{i,j}$ is the probability that the i th sample is predicted to be j.

The weights W are decided based on the relative frequencies of the classes and their importance in the segmentation task. Higher weights are assigned to tobacco shreds (especially those with fewer samples), which ensures that the model is more sensitive to these classes, improving classification accuracy. The background class is given a lower weight to avoid overfitting to the majority class. The overall weight design follows an inverse frequency approach, with manual adjustments for specific class characteristics to balance the model’s performance across all classes.

#### 3.1.2 Applying the cosine annealing algorithm to optimize the learning rate adjustment strategy

During model training, the model parameters are initialized to random values. After the point cloud data are input into the model, the predicted values are calculated through forward propagation of the neural network. These values are compared with the true labels, and the loss function is calculated to measure the accuracy of the model’s predictions. The gradient of the loss function with respect to each model parameter is calculated using the backpropagation algorithm. The gradient represents the rate of change of the loss function with respect to the parameters, guiding how the parameters should be adjusted to reduce the loss. The optimizer then updates the model parameters based on the calculated gradients.

These steps are repeated over multiple epochs until the model reaches the predetermined number of training epochs. The learning rate controls the size of each parameter update, affecting the model’s convergence speed and final performance. The original learning rate adjustment strategy involved multiplying the learning rate by a decay rate coefficient at fixed steps. However, this fixed-step reduction may not adapt to dynamic changes during the training process. Lowering the learning rate too early may cause the model to stop converging before reaching the optimal solution, affecting its final performance.

Therefore, we modified the learning rate adjustment strategy by applying the cosine annealing algorithm to adjust the learning rate to improve the overall performance. The core idea of the cosine annealing algorithm is to use the shape of a cosine function to adjust the learning rate. At the beginning of training, a larger learning rate is used, which helps the model approach the optimal solution more quickly as it allows the parameters to move more significantly in the parameter space. As training progresses, the learning rate gradually decreases, allowing the model to finetune the parameters as it approaches the optimal solution, thereby improving the model’s convergence speed and effect. The formula for the cosine annealing algorithm is as follows:


(4) $$\eta_{t}=\eta_{\text{min}}+\frac{1}{2}\left(\eta_{\text{max}}-\eta_{\text{min}}\right)\left(1+\text{cos}\left(\frac{{\text{T}}_{\text{i}}}{{\text{T}}_{\text{restart}}}\pi \right)\right)$$

**Figure 4:**
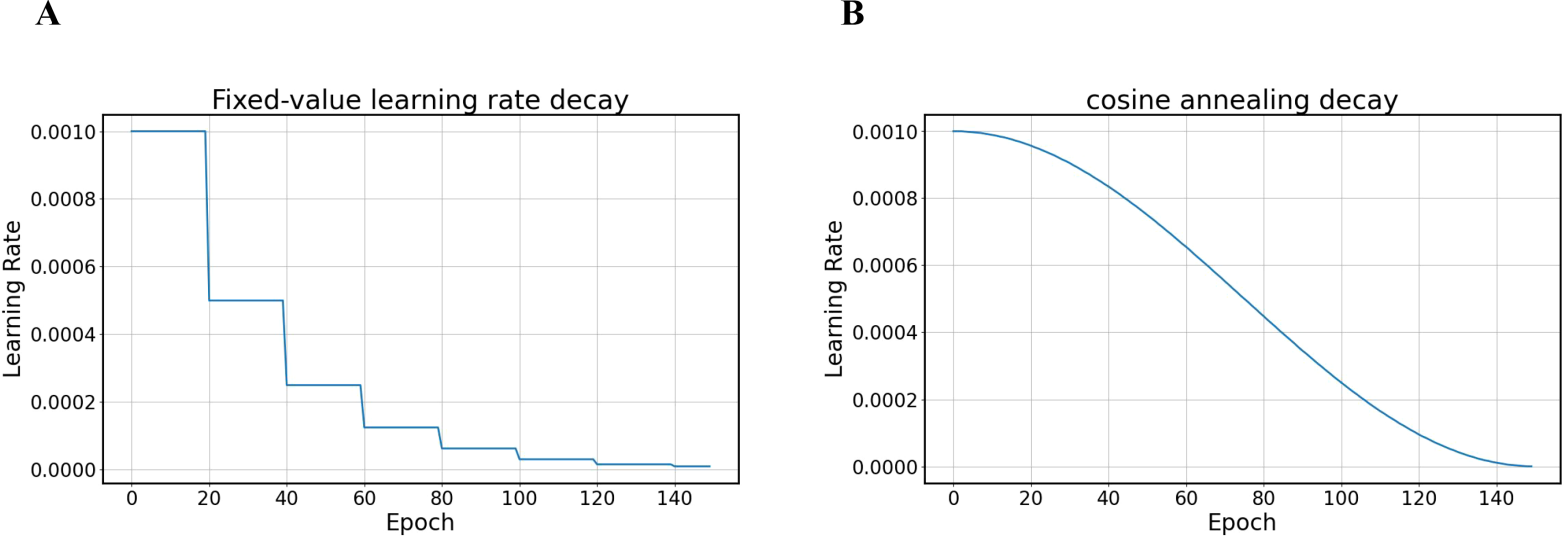
Comparison of learning rate decay methods **(A)** fixed-value decay **(B)** cosine annealing decay.

In Equation 5, $\eta_{t}$ is the learning rate of the step (or epoch). $\eta_{\text{min}}$ is the minimum learning rate in cosine annealing, which is usually set to a very small value, and $\eta_{\text{max}}$ is the maximum learning rate in cosine annealing, which is applied before each restart. ${\text{T}}_{\text{i}}$ is the number of epochs (or steps) that have been carried out, which is incremented from 1. ${\text{T}}_{\text{restart}}$ is the number of cosine annealing periods, i.e., after how many epochs is a restart performed. The detailed learning rate decay is shown in **Figure 4**.

#### 3.1.3 Improving the multi-scale grouping method of PointNet++

In PointNet++, the farthest point sampling (FPS) algorithm is used to select a subset of the point cloud to ensure that the sampled points are uniformly distributed in the point cloud data. However, the ball query method used in the original network has shortcomings with respect to the grouping process: the size of its result set is affected by the radius selection. If the radius is too large, it may lead to an excessive number of neighboring points and increase the computational complexity; conversely, if it is too small, important neighboring points may be missed. Tobacco shred features tend to be more subtle and complex in shape. KNN can accurately select a fixed number of nearest neighbor points in a local range, thus improving the model’s detail recognition and classification or part-segmentation accuracy. The use of KNN ensures that the feature dimension of each local region is consistent during feature extraction, which helps in training and optimizing the network. Meanwhile, because of the uniform density of the acquired point cloud data, KNN can select a fixed number of neighboring points for each sampling point to effectively capture local features. In point clouds with complex geometries, KNN is also better at recognizing detailed features and enhancing the understanding of complex shapes.

To further improve the performance of PointNet++, this study dynamically adjusted the k-value of KNN according to the local features of the point cloud, so that it captures enough feature information at different scales. This improvement not only reduces the computational complexity, but also prevents the omission of feature information and enhances the model’s ability to handle complex geometries.

### 3.2 3D size measurement method for mixed tobacco shreds

Conventional tobacco shred size measurement methods typically use 2-D images to calculate the length and width of tobacco shreds. However, because tobacco shreds are soft, they may bend or curl when spread out on a plane, making it challenging to measure their actual length from 2-D images. Specifically, the image method involves photographing the object with a camera, then processing and analyzing the images to measure the object’s length and width. During this process, the measurement result from the image method is the projection length of the object on the 2-D plane rather than the actual length in 3-D space. At the same time, 2-D measurement methods cannot measure tobacco shred thickness. However, by scanning the point cloud data of the upper and lower sides of the tobacco shreds, the point clouds can be merged to approximate their overall outline. Then, the improved PointNet++ algorithm can be used to segment the tobacco shreds, obtaining the position information of each shred. Using the position information, the 3-D size of the tobacco shreds can be calculated. This study proposes a DTC algorithm to calculate the true length, width, and thickness of the tobacco shreds by mapping from 3-D to 2-D and then back from 2-D to 3-D.

#### 3.2.1 Point cloud registration

Because there are three-axis motion and installation accuracy issues in the point cloud data acquisition system, the original point cloud scan data may appear misaligned within the same coordinate system. Therefore, the data from the upper and lower sides of the stage must be registered. Point cloud registration algorithms align point cloud data acquired from different viewpoints to construct a complete 3-D scene or achieve more accurate target recognition. Point cloud registration algorithms require sufficient overlapping regions between datasets. These overlapping regions contain the same object or surface features, allowing the algorithm to find matching points and align them. However, the point cloud data acquired in this experiment only include the upper and lower surface layers of the scanned object, with no overlapping regions. Therefore, traditional point cloud registration algorithms are insufficient for providing realistic thickness values for the scanned data from both sides. We propose a method for point cloud data registration based on standard gauge blocks.

In this experiment, a standard gauge block with a length of 30 mm, a width of 9 mm, a thickness of 0.5 mm, and a limit deviation of 0.12 μm was used to calibrate and verify the accuracy of the point cloud data. First, the standard gauge block was placed on the stage and scanned along with the tobacco shreds, resulting in scan data for both upper and lower surfaces. After preprocessing the point cloud data, the standard gauge block was segmented, and the center point, two midpoint coordinates along the length, and two midpoint coordinates along the width were used as reference points. The registration formulas are as follows:


(5) $$x^{\prime }=x+\frac{1}{5}\sum_{i=1}^{5}\text{Δ}x_{i}$$

(6) $$y^{\prime }=y+\frac{1}{5}\sum_{i=1}^{5}\text{Δ}y_{i}$$

(7) $$z^{\prime }=z+\frac{1}{5}\sum_{i=1}^{5}\text{Δ}z_{i}$$

In Equations 5–7, x, y, and z represent the upper-side point cloud data, $\text{Δ}x_{i}$ , $\text{Δ}xy_{i}$ , $\text{Δ}z_{i}$ are the differences of the five sets of coordinate points above and below the block, and $x^{\prime }$ , $y^{\prime }$ and $z^{\prime }$ are the lower-side calibrated point cloud data.

#### 3.2.2 Tobacco shred length and width algorithm

**Figure 5:**
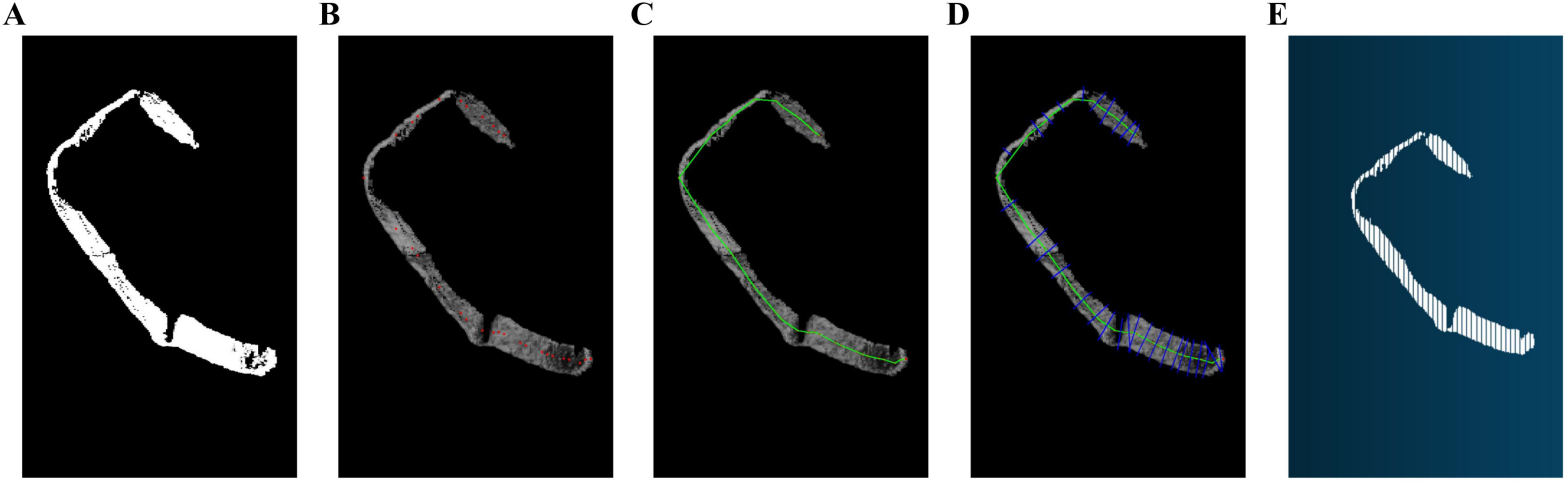
Flow of the length and width algorithm: **(A)** 2D image of tobacco shred, **(B)** Finding the center of the skeleton, **(C)** Tobacco Shred Skeleton Seeking Excellence, **(D)** Width coordinate positioning, **(E)** Point cloud traceability.

After point cloud registration, accurate point cloud data of the tobacco shreds can be obtained. However, due to the complex physical characteristics of mixed tobacco shreds, such as bending or twisting, it is cumbersome to calculate their three dimensions using raw point cloud data alone. The DTC algorithm was used to calculate the true length and width of the tobacco shreds, greatly reducing the algorithm’s complexity. The specific steps of the algorithm are as follows:

• Obtain a 2-D image of the tobacco shreds. The original point cloud data obtained through scanning are presented as a matrix, with the horizontal and vertical axes representing the scanning coordinates and the values within the coordinates representing the scanning height data. Since the point cloud height data for the tobacco shreds are concentrated between 26,000 and 28,000, and any data outside this range correspond to background data, mapping the height data within this range to a grayscale image with pixel values ranging from 0 to 255 yields the 2-D projection image of the tobacco shred.

• Obtain the position coordinates of the 2-D image of the tobacco shreds. After segmentation using an improved PointNet++ model, the tobacco shred data in the 3-D point cloud are obtained. To map this data onto the 2-D image, only the x and y coordinates of the outermost contour of the tobacco shred are needed to determine its position. The mapped 2-D image is shown in **Figure 5A**.

• Extract the skeleton. After obtaining the contour position of the tobacco shred, the data are divided along the main trunk in the vertical direction into a certain number of segments. Each segment is then analyzed individually. When a segment contains a single tobacco shred, its center point is calculated. As shown in the lower half of **Figure 5B**, where there is only one segment in the horizontal direction, only one center point exists in that direction. If multiple segments are obtained, the center points of each tobacco shred in the contour are calculated. In the upper half of **Figure 5B**, two tobacco shreds are found, and their respective center points must be calculated. After obtaining the center point coordinates of the tobacco shred in each segment, the coordinates are analyzed to obtain the optimal order in which to connect them to a main skeleton, as shown in **Figure 5C**. Specifically, the first center point is chosen as the starting point, and the remaining points are traversed to find the closest point. This point is then connected to the starting point, and the next closest point is found and connected. This process continues until all center points are connected.

• Calculate the length. The skeleton coordinates (x, y) are mapped back to the point cloud data (**Figure 5E**) to obtain the corresponding z-coordinate. The length is calculated by determining the distance between two points (x, y, z) in space. The calculation formula is as follows:


(8) $$l=\sum_{i=1}^{N-1}\sqrt{{\left(x_{\text{i}}-x_{\text{i}-1}\right)}^{2}+{\left(y_{\text{i}}-y_{\text{i}-1}\right)}^{2}+{\left(z_{\text{i}}-z_{\text{i}-1}\right)}^{2}}$$

• Calculate the width. After obtaining the tobacco shred contour’s center points and connecting them optimally to form the skeleton, a line segment is formed by connecting each pair of neighboring center points. A perpendicular line is drawn to this segment, intersecting the tobacco shred contour at two points, as shown in **Figure 5D**. By mapping these intersection points back to the point cloud data (**Figure 5E**), the real 3-D average and maximum widths of the tobacco shred can be calculated. The specific calculation formulas are as follows:


(9) $$w_{a}=\frac{1}{N-1}\sum_{c=1}^{N-1}\sum_{j=1}^{S}\sqrt{{\left(x_{\text{c}}-x_{\text{j}}\right)}^{2}+{\left(y_{\text{c}}-y_{\text{j}}\right)}^{2}+{\left(z_{\text{c}}-z_{\text{j}}\right)}^{2}}$$

(10) $$w_{m}=max_{c=1,2,\ldots ,N}\left(\sum_{j=1}^{S}\sqrt{{\left(x_{\text{c}}-x_{\text{j}}\right)}^{2}+{\left(y_{\text{c}}-y_{\text{j}}\right)}^{2}+{\left(z_{\text{c}}-z_{\text{j}}\right)}^{2}}\right)$$

In Equations 9, 10, $w_{a}$ is the average width of the filament, $w_{m}$ is the maximum width of the filament, $x_{\text{c}}$ , $y_{\text{c}}$ , and $z_{\text{c}}$ are the midpoints of the two center points of the skeleton, and $x_{\text{j}}$ , $y_{\text{j}}$ , and $z_{\text{j}}$ are the intersections of the perpendicular lines with the filament contour. N is the number of skeleton center coordinates. The flowchart of the length and width algorithm is shown in **Figure 5**.

#### 3.2.3 Tobacco shred thickness measurement algorithm

To measure the overall average thickness of the shred, the distance between a point on the upper surface of the tobacco shred and the corresponding point on the lower surface is calculated. The average thickness value T is then calculated by subtracting or adding the corresponding height difference between the upper and lower surfaces of the tobacco shred and the standard gauge block. The standard gauge block’s thickness is denoted as H, and the algorithm’s specific steps are as follows:

- Segment the point cloud using the improved model to obtain the point cloud sets of the upper and lower surfaces of different tobacco shreds.
- Calculate the distance between each point on the upper surface of the tobacco shred and each point on the upper surface of the standard gauge block to obtain the average height difference H1. Similarly, calculate the distance between each point on the lower surface of the tobacco shred and each point on the lower surface of the standard gauge block to obtain the average height difference H2.
- Depending on the positional relationship between the tobacco shred and the standard gauge block, two cases are considered:

When the average height of the upper surface of the tobacco shred is lower than the upper surface of the standard gauge block, the tobacco shred’s thickness is calculated as follows:


(11) T=H−H1−H2

When the average height of the upper surface of the tobacco shred is higher than the upper surface of the standard gauge block, the tobacco shred’s thickness is calculated as follows:


(12) T=H+H1−H2

In Equations 11, 12, H1 and H2 are the average distances between the points, so the values are both greater than 0. The method for calculating the tobacco shred’s thickness value T involves defining two reference planes and calculating the thickness based on the distance between them. The principle of the thickness algorithm is shown in **Supplementary Figure 4**.

To further clarify the principle of the tobacco shred thickness measurement algorithm, an example using actual tobacco shreds and reference block data is presented. **Supplementary Figure 5A** shows the top view of the tobacco shred and the reference block. **Supplementary Figure 5B** depicts the scanned point cloud of the upper surface, where a certain distance below the reference block represents its thickness H. This becomes more apparent when comparing with the point cloud scanned from the bottom surface, as shown in **Supplementary Figure 5C**.

## 4 Results and analysis

### 4.1 Experimental hardware and software platform and parameter settings

This experiment was conducted on a Windows 10 platform equipped with an NVIDIA GeForce RTX 4090 GPU (24GB VRAM), an Intel Core i9-14900KF CPU (3.2GHz), and 64GB of memory. The model was implemented and trained using the PyTorch deep learning framework with Python, utilizing CUDA for parallel computation. The development environment was PyCharm. The Python version used was 3.9, and the CUDA version was 12.4. For each sample, 4096 points were randomly selected from the original point cloud data for network input. During training, the model was trained for 150 iterations with a batch size of 16.

### 4.2 Model evaluation metrics

Objective performance metrics such as Mean Intersection over Union (MIoU), Precision (Pre), Recall (Rec), and F1-Score are typically used to evaluate the performance of a deep learning-based 3-D semantic segmentation network model.

MIoU is one of the most commonly used metrics for the semantic segmentation of point clouds and measures the average prediction accuracy for each category. The formula is as follows:


(13) $$mIoU=\frac{1}{\text{N}}\sum_{\text{i}=1}^{\text{N}}\frac{{\text{TP}}_{i}}{{\text{TP}}_{i}+{\text{FP}}_{i}+{\text{FN}}_{i}}$$

Pre and mPre represent the proportion of points that the model predicts as belonging to a category that actually belong to that category and the average precision rates for each category, respectively. The formula is as follows:


(14) $$Pre_{i}=\frac{{\text{TP}}_{i}}{{\text{TP}}_{i}+{\text{FP}}_{i}}$$

(15) $$mPre=\frac{1}{N}\sum_{i=1}^{N}\frac{{\text{TP}}_{i}}{{\text{TP}}_{i}+{\text{FP}}_{i}}$$

mRec represents the average of the proportion of samples correctly predicted as positive classes by the model. The formula is as follows:


(16) $$m\text{Rec}=\frac{1}{N}\sum_{i=1}^{N}\frac{{\text{TP}}_{i}}{{\text{TP}}_{i}+{\text{FN}}_{i}}$$

The F1-Score is the average of the reconciled mean of precision and recall for each category, which is used to balance the two metrics and is calculated using the following formula:


(17) $$F_{1}=\frac{1}{N}\sum_{i=1}^{N}2\times \frac{{\text{Pre}}_{i}\times {\text{Rec}}_{i}}{{\text{Pre}}_{i}+{\text{Rec}}_{i}}$$

In Equations 8–12, i represents the different categories, N is the number of categories, and TP_i is the true positive instances of each category, i.e., the number of points the model correctly predicts as belonging to the positive category. FP_i is the false positive instances of each category, i.e., the number of points the model incorrectly predicts as belonging to the positive category. FN_i is the false negative instances of each category, i.e., the number of points the model incorrectly predicts as belonging to the negative category. The above four metrics are used to evaluate the point cloud semantic segmentation model’s performance. Because of the large amount of data in the background point cloud, the evaluation metrics will have an impact on the four tobacco targets, so only the four tobacco categories are counted when calculating the average evaluation metrics.

### 4.3 Data augmentation comparison experiment

**Figure 6:**
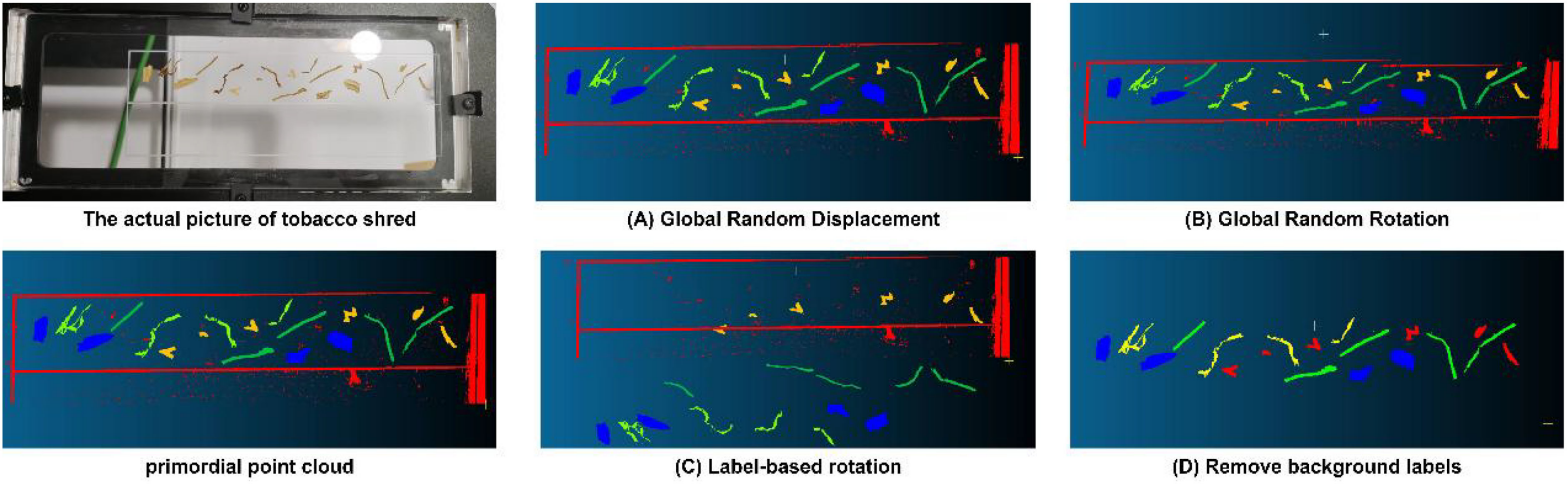
The actual picture of tobacco shreds primordial point cloud with various transformations and label-based operations: **(A)** Global Random Displacement, **(B)** Global Random Rotation, **(C)** Label-based Rotation, **(D)** Remove background labels.

This experiment used four data augmentation methods: global random displacement, global random rotation, label-based rotation, and background label removal. **Figure 6** displays the tobacco shred’s physical image and the point cloud changes under different augmentation methods. Training was performed by combining the original data with the new datasets generated by these four augmentation methods, resulting in the outcomes shown in **Table 2**. The results indicated that global random displacement and background label removal significantly improved the MIoU and F1 combined metrics, and global random rotation also enhanced the model’s performance. However, the label-based rotation method had a negative impact on model performance. Therefore, in subsequent experiments, we used three data augmentation methods: global random displacement, global random rotation, and background label removal. The amount of data generated by each method was the same as the original data, ultimately resulting in a dataset size four times larger than the original dataset.

**Table 2:** Results of the average indicator for the data enhancement methodology.

GRD	GRR	LBR	RBL	mIoU (%)	mPre (%)	mRec (%)	$F_{1}$ (%)
				44.71	51.53	45.59	48.37
√				71.93	80.38	79.34	79.85
	√			50.08	59.06	55.77	57.37
		√		45.26	49.48	47.62	48.53
			√	66.52	79.52	78.56	79.03

### 4.4 Segmentation model results

#### 4.4.1 Overall testing of the improved PointNet++ network model

**Figure 7:**
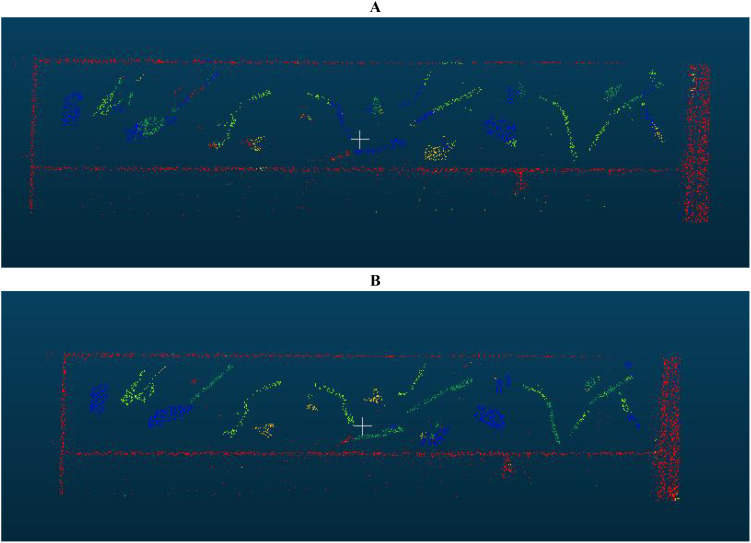
Predicted images before and after model improvement: **(A)** Image predicted by PointNet++; **(B)** Image predicted by improved PointNet++.

The improved PointNet++ network model was trained and tested for 150 epochs under the configuration and parameter settings described in Section 4.1 combined with the data augmentation methods described in Section 4.3. **Figure 7** presents the results, demonstrating significant improvements in segmentation performance and supporting further processing of the segmented point cloud in subsequent algorithms.

**Table 3** compares the performance of the network before and after the improvement. The improved model showed a significant improvement in training results on the same dataset. Specifically, the average precision increased from 0.8427 to 0.9498, the average intersection over union (mIoU) increased from 0.7511 to 0.9181, the average recall increased from 0.8363 to 0.9766, and the F1-Score increased from 0.8395 to 0.9631. This indicates significant improvements in recall, overall performance, accuracy, and segmentation precision, resulting in higher accuracy and consistency. Additionally, the training time for the PointNet++ network was 4,554 seconds, while for the improved network’s it was 4,229 seconds. The prediction time for each point cloud file was 0.326 seconds, meeting the requirements of practical engineering applications.

**Table 3:** Improved Point Net++ network model performance.

Model	mPre (%)	mIoU (%)	mRec (%)	$F_{1}$ (%)	Time (s)
PointNet++	84.27	75.11	83.63	83.95	4554
Improved PointNet++	95.13	92.46	97.68	96.39	4212

#### 4.4.2 Performance testing of the improved loss function

We validated the improved loss function described in Section 3.2.2 by comparing the performance of the original loss function with that of the improved weighted cross-entropy loss function. In the weighted cross-entropy loss function, different weights need to be set for each class. Since background point cloud data are large and easy to learn, the weight of the background point cloud label must be reduced, while the weights of the four tobacco shred labels must be increased. For this purpose, we set different weight combinations and implemented step-by-step increases in the weights of the tobacco shred classes. The label order is: w_0 [1,1,1,1,1], w_1 [2,2,2,2,1], w_2 [3,3,3,3,1], w_3 [4,4,4,4,1], w_4 [5,5,5,5,1], w_5 [10,10,10,10,1], w_6 [20,20,20,20,1], w_7 [1,7,1,7,1], w_8 [2,6,2,6,1], and w_9 [3,5,3,5,1].We tested the effect on performance until extreme weights were reached. Meanwhile, according to the initial model’s varying precision rates for each type of tobacco shred, we adjusted the weights between different tobacco shreds to select the best parameters.

**Table 4** compares the performance of the network with the weighted cross-entropy loss function under different weights, and the network with weighted cross-entropy loss function showed improved overall improved performance compared with w-0. After experimental comparison, it was found that the model parameters of w_3, with a weighting ratio of [4,4,4,4,1], were the highest of the three indexes, and that the leaf filaments and the expanded filaments—also known as 1,3 labels—were lower than the parameters of the other labels. Therefore, three sets of controlled experiments were conducted to determine whether the different weights within the tobacco filaments could improve the overall parameters of the model. After performing the controlled experiments, it was found that improving the two labels that originally had low weights instead led to a decrease in the model’s overall performance, and w-3 was ultimately chosen as the optimal weighting parameter.

**Table 4:** Impact of weighted cross-entropy loss on model performance with different weights.

Weight categories	mPre (%)	mIoU (%)	mRec (%)	$F_{1}$ (%)	Time (s)
w-0	84.27	75.11	83.63	83.95	4554
w-1	87.05	79.61	87.94	87.49	4512
w-2	89.01	82.78	90.93	89.96	4619
w-3	89.27	83.50	91.88	90.56	4883
w-4	88.21	82.89	91.20	89.68	4836
w-5	87.72	81.79	91.78	89.70	4792
w-6	86.21	80.60	92.08	89.05	4378
w-7	81.84	73.10	85.97	83.85	4573
w-8	87.24	81.12	90.55	88.86	4586
w-9	86.50	80.33	89.19	87.82	4598

#### 4.4.3 Performance testing of the cosine annealing algorithm optimized learning rate

The effect of learning rate optimization using the cosine annealing algorithm on model performance was verified on the basis of the improvements detailed in Section 4.4.2, comparing the network performance of both models with the above-mentioned parameter configurations. The network subjected to the intervention of learning rate optimization using the cosine annealing algorithm is called cos-w-3-PointNet++.

**Table 5** shows that optimizing the learning rate using the cosine annealing algorithm on the basis of the improved cross-entropy loss function outlined in Section 4.4.2 does not greatly improve the average precision rate of the improved PointNet++ network. However, the average cross-merge ratio is increased from 83.50% to 86.84% and the average recall rate is increased from 91.88% to 95.98%. The F1-Score thus improves from 90.56% to 93.21%, and the model’s training time is also reduced.

**Table 5:** Comparison of the effect of cosine annealing algorithms on performance metrics.

Network Models	mPre (%)	mIoU (%)	mRec (%)	$F_{1}$ (%)	Time (s)
w-3-PointNet++	89.27	83.50	91.88	90.56	4883
cos-w-3-PointNet++	90.59	86.84	95.98	93.21	4617

#### 4.4.4 Performance testing of the improved PointNet++ multi-scale grouping method

**Figure 8:**
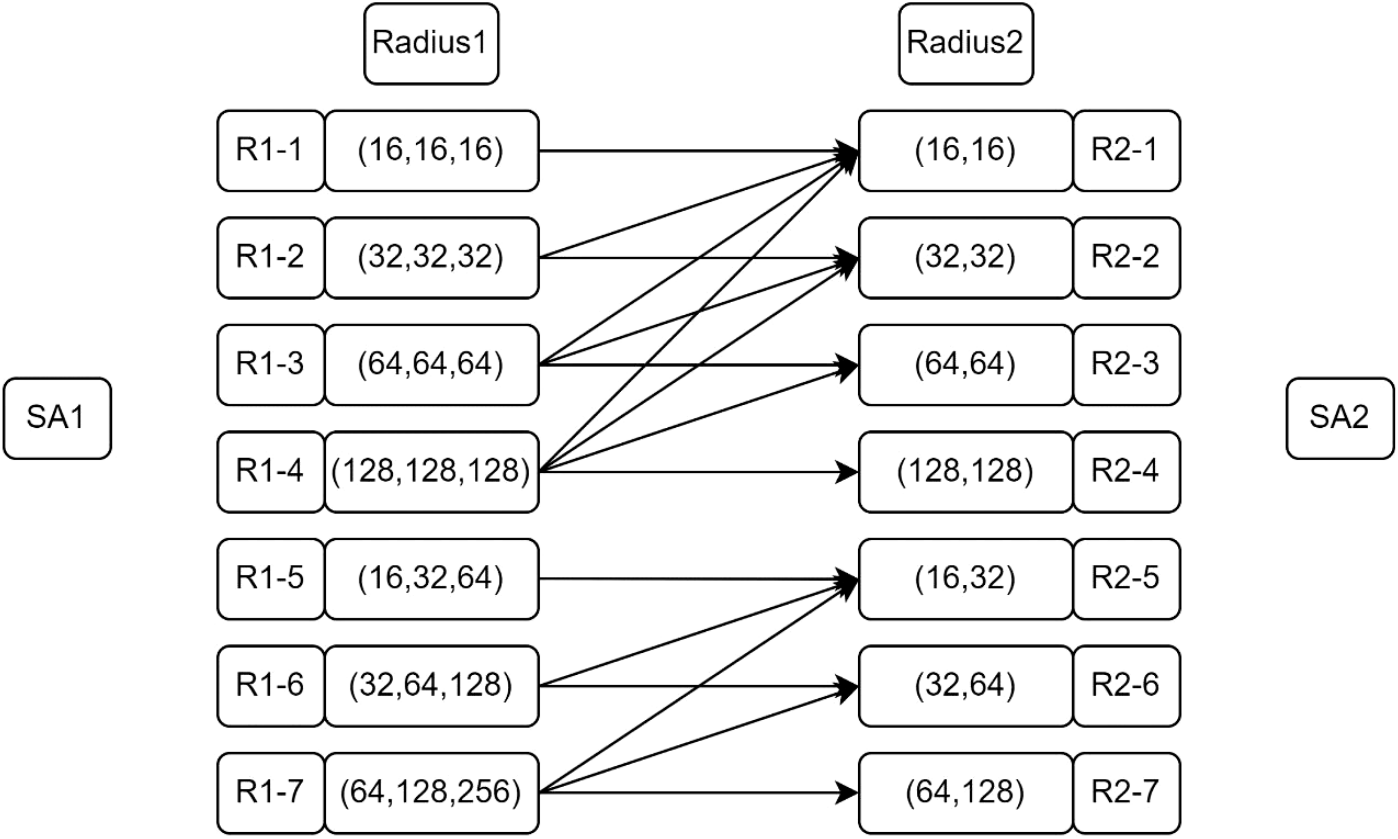
Group splicing methods at different radii.

The improved PointNet++ multi-scale grouping method described in Section 3.2.4 was validated based on the improvements described in Section 4.4.3. The performance of the different grouping methods was tested by combining KNN with PointNet++ to process complex the geometrical point cloud data of tobacco shreds. The results showed that the improved multi-scale grouping method significantly enhanced the model’s performance. The results indicated that the model’s performance under different radii of grouping splicing exhibited various behaviors. The splicing method at different radii is shown in **Figure 8**.

The improved multi-scale grouping method improves the performance of the model to a large extent on the basis of the first grouping sampling 512 points and the second grouping sampling 128 points, combined with the different K values of KNN to sample the point cloud for the second time. The optimal model parameters were found through the different sub-splices presented in **Table 6**, and it can be seen that the model’s performance under the different radius splices has different performances. When the K value obtains R1-2 -R2-1, although mPre and mIoU are lower compared with R1-5-R2-R and R1-6-R2-5, the mRec and F1 scores reach 97.68% and 96.39%, respectively, and the training time is also the fastest of all the other model parameters. Therefore, the splicing combination of R1-2-R2-1 was chosen as the final model parameter.

**Table 6:** Comparison of the effects of different splicing relationships on performance metrics.

Network Models	mPre (%)	mIoU (%)	mRec (%)	$F_{1}$ (%)	Time (s)
cos-w-3-PointNet++	90.59	86.84	95.98	93.21	4617
R1-1-R2-1	94.59	91.63	97.23	95.89	4215
R1-2-R2-1	95.13	92.46	97.68	96.39	4212
R1-2-R2-2	95.00	91.81	97.66	96.31	4239
R1-3-R2-1	94.60	91.50	97.34	95.95	4422
R1-3-R2-2	94.59	91.78	97.30	95.92	5208
R1-3-R2-3	93.73	90.93	96.32	95.01	5127
R1-4-R2-1	94.83	92.62	97.45	96.12	4857
R1-4-R2-2	94.62	92.04	96.81	95.70	4747
R1-4-R2-3	93.66	90.83	96.25	94.94	4774
R1-4-R2-4	93.52	90.81	95.90	94.70	5322
R1-5-R2-5	95.17	92.84	97.52	96.33	4519
R1-6-R2-5	95.91	92.77	96.51	95.91	4644
R1-6-R2-6	94.72	92.19	97.19	95.93	4758
R1-7-R2-5	94.64	92.14	97.24	95.92	4986
R1-7-R2-6	94.46	91.82	96.89	95.66	5761
R1-7-R2-7	93.63	90.21	96.25	94.93	5250

### 4.5 Analysis of 3D size measurement results

Ten samples of each of the four types of tobacco silk (Cut Stem, Tobacco Silk, Reconsitituted Tobacco Shred, Expanded Tobacco Silk) were randomly selected and placed in a humidity chamber at a constant temperature for 24h. Five standard measurement blocks were used as control materials. Manual measurement was carried out by stretching and flattening the filaments using vernier calipers, while the traditional 2-D algorithmic and our 3-D measurement methods were used by placing the tobacco shreds horizontally on a carrier table.

**Figure 9:**
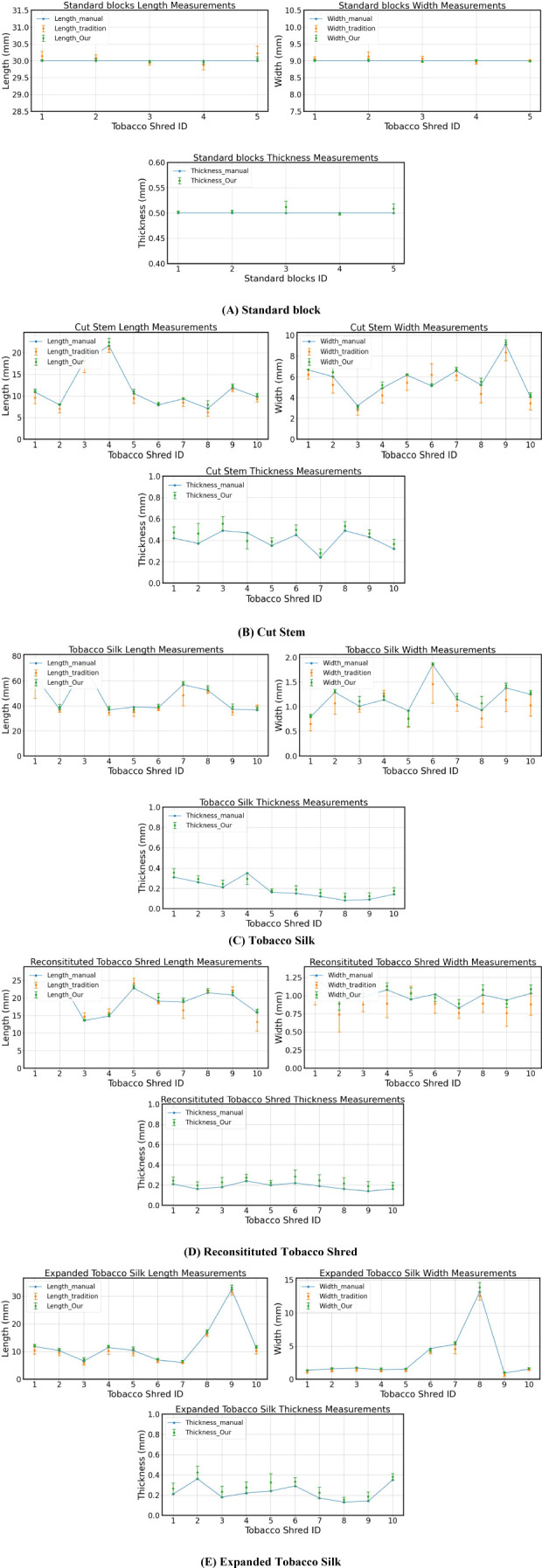
Comparison of measurement results: **(A)** Standard blocks; **(B)** Cut Stem; **(C)** Tobacco Silk; **(D)** Reconsitituted Tobacco Shred; **(E)** Expanded Tobacco Silk.

The calculation results are shown in **Figure 9**; the length and width of the standard block and the four kinds of tobacco were measured using three different methods, namely manual measurement, the traditional 2-D algorithm, and the 3-D algorithm proposed in this paper. Because the traditional 2-D algorithm cannot calculate the thickness value, this value was only compared with the manual measurement.

When measuring length and width, the traditional 2-D algorithm can only capture the projection of the tobacco when photographing curved tobacco, and there is no way to obtain its height information. Therefore, the traditional 2-D algorithm can only measure regular and non-curled tobacco, and the experimental materials selected in this paper have a certain degree of curvature. The 3-D algorithmic measurement method proposed in this paper is significantly better than the traditional 2-D algorithmic measurement method, as can be seen in **Supplementary Tables 1**, **2**, which show that the relative error of our 3-D method is generally less than that of the traditional 2-D algorithmic measurement. The effect of our 3-D method is also is significantly better than that of the ordinary 2-D algorithm in measuring curled tobacco. The relative errors of the length of the tobacco for both methods are all less than 5%, and the relative errors of the width are all less than 7%.

**Supplementary Table 3** shows that, by comparing the manual measurement results and those of the 3-D algorithm, the relative error of the tobacco shred thickness value is generally larger. After experimentation, because the texture of the tobacco shred is soft, using vernier calipers for measuring its thickness will lead to compression, so using the 3-D algorithm for measurement is worth achieving results that are generally larger than the manual measurement value as it can measure tobacco shreds accurately without touching them. Overall, our DTC algorithm has a smaller relative error than that of the traditional 2-D algorithm when calculating length and width. Although the relative error when measuring thickness is larger compared to that of the manual measurement, our algorithm can achieve non-contact measurement of the tobacco shred’s thickness, and by using a standard block of measurements to validate the thickness algorithm, the final relative error can reach 1.12%.

## 5 Conclusion

To solve the current problem of the difficulty in measuring the 3-D dimensions of mixed tobacco, this paper proposes a measurement method based on the results of tobacco point cloud segmentation. The PointNet++ model is improved on the basis of PointNet++, and the weighted cross-entropy loss function is used to give higher weight to the minority class samples, which improves the model’s ability to identify the minority class; the learning rate is dynamically adjusted by the cosine annealing algorithm, which enables the model to approach the optimal solution quickly at the initial stage, and finely adjusts it at the later stage, which optimizes the model’s training effect; the improved multi-scale grouping method Combined with the KNN algorithm, it ensures the stability and consistency of feature extraction at different scales, and improves the model’s ability to understand and recognize complex geometric shapes. Together, these improvements enable the model to complete the segmentation task more accurately and efficiently when dealing with tobacco point cloud data.

With the help of the tobacco segmentation model, the measurement of the three-dimensional dimensions of the blended tobacco was achieved by designing a DTC algorithm. The experimental results show that the length and width measurements are closer to the manual measurements than the traditional two-dimensional size measurement methods, and the relative error is smaller. Moreover, non-contact measurement of tobacco thickness can be realized.

Conventional methods of measuring the dimensions of tobacco filaments usually rely on two-dimensional images, and the actual length measurement is complicated by the softness of the filaments, which may bend or curl on a flat surface. The 2D image method measures length and width as a two-dimensional projection through camera capture and image processing and does not capture the thickness of the tobacco. The DTC algorithm, on the other hand, uses point cloud data from the top and bottom sides of the scanned tobacco filaments, which can be merged to approximate the overall contour of the filaments and segmented by applying the improved PointNet++ algorithm to obtain the positional information of each filament. The algorithmic process of finding and calculating the point position is simplified by mapping the point cloud data of the tobacco filaments from 3D to 2D, and then from 2D back to 3D to calculate the true length, width and thickness of the filaments.

This DTC algorithm based on point cloud segmentation results has significant advantages not only for 3D dimensional measurements of blended tobacco but can also be widely applied to measurements of other soft materials, such as textiles, films and food products. The flexibility of this approach allows it to effectively handle the complex shapes and properties of different materials, thus improving the accuracy and efficiency of the measurements.

In the future, with the continuous advancement of deep learning and point cloud processing technologies, DTC algorithms may be applied in more fields to promote the further development of industrial automation and intelligent manufacturing, especially in quality control and material inspection, to enhance overall production efficiency and product quality.

## Data Availability

The original contributions presented in the study are included in the article/**Supplementary Material**. Further inquiries can be directed to the corresponding authors.

## References

[B1] Acuña A. M. (2017). The framework convention on tobacco control of the World Health Organization. Rev. Chil. Enferm. Respir. 33, 180–182.

[B2] Bharati P. Pramanik A. (2020). “Deep learning techniques—R-CNN to mask R-CNN: A survey,” in Computational Intelligence in Pattern Recognition. Eds. Das A. K. Nayak J. Naik B. Pati S. K. Pelusi D. (Springer Singapore, Singapore), 657–668. doi: 10.1007/978-981-13-9042-5_56

[B3] He K. Zhang X. Ren S. Sun J. (2016). “Deep residual learning for image recognition,” in 2016 IEEE Conference on Computer Vision and Pattern Recognition (CVPR), Las Vegas, NV, USA: IEEE. 770–778. doi: 10.1109/CVPR.2016.90

[B4] Jia K. Niu Q. Wang L. Niu Y. Ma W. (2023). A new efficient multi-object detection and size calculation for blended tobacco shreds using an improved YOLOv7 network and LWC algorithm. Sensors 23, 8380. doi: 10.3390/s23208380 37896474 PMC10610831

[B5] Jiang H. Learned-Miller E. (2017).Face detection with the faster R-CNN. Available online at: https://ieeexplore.ieee.org/abstract/document/7961803/ (Accessed September 26, 2024). IEEE.

[B6] Li X. Wang W. Wu L. Chen S. Hu X. Li J.. (2020). Generalized focal loss: Learning qualified and distributed bounding boxes for dense object detection. Adv. Neural Inf. Process. Syst. 33, 21002–21012.

[B7] Liu J. Niu Q. Jin Y. Chen X. Wang L. Yuan Q. (2022). Research on tobacco shred image recognition method based on efficient channel attention mechanism and multi-scale feature fusion. Henan Agric. Sci. 51, 145–154. doi: 10.15933/j.cnki.1004-3268.2022.11.017

[B8] Lu Y. Chen Y. Zhao D. Chen J. (2019). “Graph-FCN for image semantic segmentation,” in Advances in Neural Networks – ISNN 2019. Eds. Lu H. Tang H. Wang Z. (Springer International Publishing, Cham), 97–105. doi: 10.1007/978-3-030-22796-8_11

[B9] Lu J. Guo H. Jia X. Wu J. Chen X. (2024). Dual fusion network for semantic segmentation of point clouds. Optics Lasers Eng. 177, 108118. doi: 10.1016/j.optlaseng.2024.108118

[B10] Mei J. Li Z. Li J. Su Z. Xu B. Du J.. (2021). Components discrimination for formula tobacco based on hyperspectral imaging. J. Analytical Testing 40, 1151–1157. doi: 10.19969/j.fxcsxb.20110702

[B11] Niu Q. Liu J. Jin Y. Chen X. Zhu W. Yuan Q. (2022a). Tobacco shred varieties classification using Multi-Scale-X-ResNet network and machine vision. Front. Plant Sci. 13, 962664. doi: 10.3389/fpls.2022.962664 36061766 PMC9433752

[B12] Niu Q. Yuan Q. Jin Y. Wang L. Liu J. (2022b). Identification of tobacco strand types based on improved VGG16 convolutional neural network. Foreign Electronic Measurement Technol. 41, 149–154. doi: 10.19652/j.cnki.femt.2203982

[B13] Qi L. Fan X. Tang X. Liu Z. Yang L. Qiao J.. (2022). Two-section cylinder dryer’s control mode on cut tobacco properties and sensory quality of demi-slim cigarettes. Tobacco Sci. Technol. 55, 88–97. doi: 10.16135/j.issn1002-0861.2021.0273

[B14] Qi C. R. Su H. Mo K. Guibas L. J. (2017a). “Pointnet: Deep learning on point sets for 3d classification and segmentation,” in Proceedings of the IEEE conference on computer vision and pattern recognition. USA: IEEE, 652–660.

[B15] Qi C. R. Yi L. Su H. Guibas L. J. (2017b). Pointnet++: Deep hierarchical feature learning on point sets in a metric space. Adv. Neural Inf. Process. Syst. 30.

[B16] Qian G. Li Y. Peng H. Mai J. Hammoud H. Elhoseiny M.. (2022). Pointnext: Revisiting pointnet++ with improved training and scaling strategies. Adv. Neural Inf. Process. Syst. 35, 23192–23204.

[B17] Redmon J. (2016). “You only look once: Unified, real-time object detection,” in Proceedings of the IEEE conference on computer vision and pattern recognition (USA: IEEE).

[B18] Sengupta A. Ye Y. Wang R. Liu C. Roy K. (2019). Going deeper in spiking neural networks: VGG and residual architectures. Front. Neurosci. 13, 95. doi: 10.3389/fnins.2019.00095 30899212 PMC6416793

[B19] Shi Z. Yang S. Kou R. Wang Y. (2024). A fast railway track surface extraction method based on bidirectional cloth simulated point clouds. Optics Lasers Eng. 180, 108335. doi: 10.1016/j.optlaseng.2024.108335

[B20] State Tobacco Monopoly Administration (2009). YC/Z 317-2009 (China: State Tobacco Monopoly Administration).

[B21] Wang L. Jia K. Fu Y. Xu X. Fan L. Wang Q.. (2023). Overlapped tobacco shred image segmentation and area computation using an improved Mask RCNN network and COT algorithm. Front. Plant Sci. 14, 1108560. doi: 10.3389/fpls.2023.1108560 37139110 PMC10150031

[B22] Wang Z. Shi J. Wang Y. Zhao M. Zhou X. Song Z.. (2020). Influences of cut tobacco width on cut tobacco structure, physical and chemical indexes, and sensory quality of cigarettes with medium circumference. Tobacco Sci. Technol. 53, 81–88. doi: 10.16135/j.issn1002-0861.2019.0089

[B23] Wang Y. Sun Y. Liu Z. Sarma S. E. Bronstein M. M. Solomon J. M. (2019). Dynamic graph CNN for learning on point clouds. ACM Trans. Graph. 38, 1–12. doi: 10.1145/3326362

[B24] Williams C. Falck F. Deligiannidis G. Holmes C. C. Doucet A. Syed S. (2023). A unified framework for U-net design and analysis. Adv. Neural Inf. Process. Syst. 36, 27745–27782.

[B25] Wu Z. Shen C. Van Den Hengel A. (2019). Wider or deeper: Revisiting the resnet model for visual recognition. Pattern recognition 90, 119–133. doi: 10.1016/j.patcog.2019.01.006

[B26] Zhang D. (2013). The mixing ratio and uniformity of expanded tobacco and cut tobacco by fast determination of FT-NIR in primary processing. J. Anhui Agric. Sci. 41, 316–317. doi: 10.13989/j.cnki.0517-6611.2013.01.037

[B27] Zhou Y. Tuzel O. (2018). VoxelNet: End-to-End Learning for Point Cloud Based 3D Object Detection. In Proceedings of the IEEE Conference on Computer Vision and Pattern Recognition (CVPR), 4490–4499. doi: 10.1109/CVPR.2018.00472

[B28] Zhou B. Zhu W. Wang Z. Jiang P. Liu H. Li Z.. (2022). Identification of tobacco materials based on terahertz time-domain spectroscopy. Trans. Chin. Soc. Agric. Eng. 38, 310–316.

