# Supplementary figures and images for: Segmentation of tobacco shred point cloud and 3-D measurement based on improved PointNet++ network with DTC algorithm
Source: Front Plant Sci. 2025 Jan 21;15:1508449. doi: 10.3389/fpls.2024.1508449 (PMC11790634; doi:10.3389/fpls.2024.1508449)

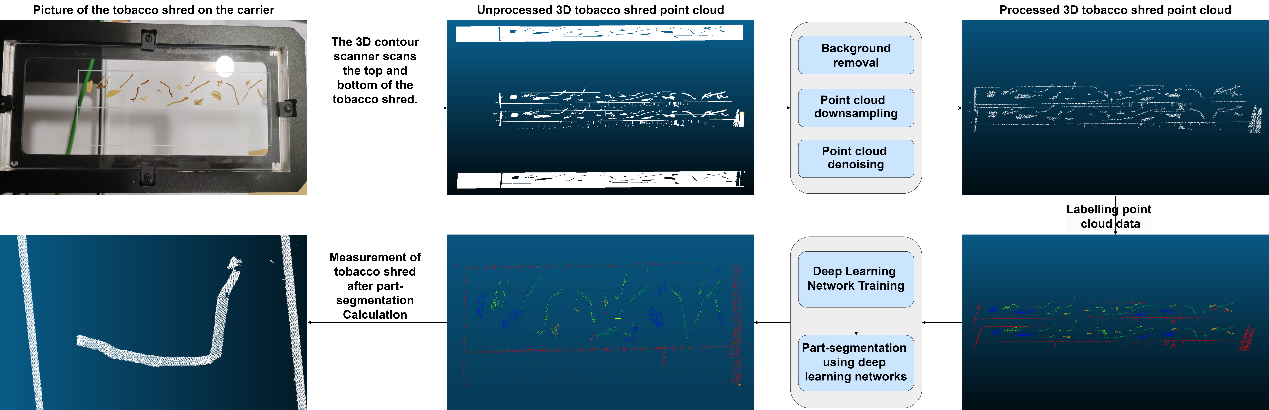

Supplement: Supplementary file 1 [file Image1.png]

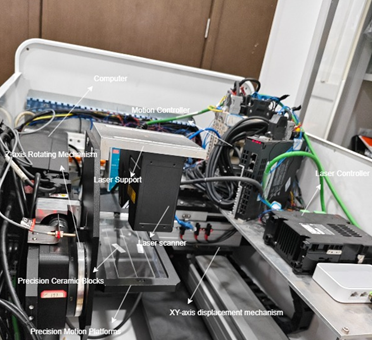

Supplement: Supplementary file 2 [file Image2.png]

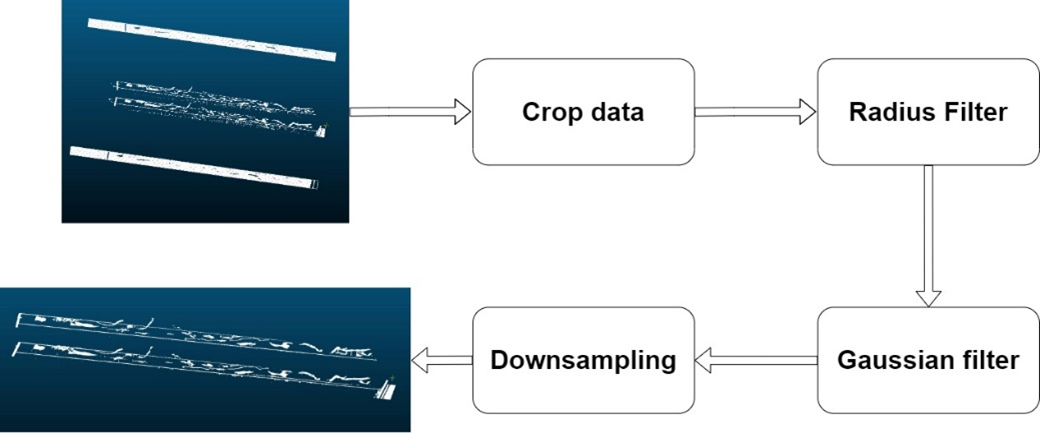

Supplement: Supplementary file 3 [file Image3.png]

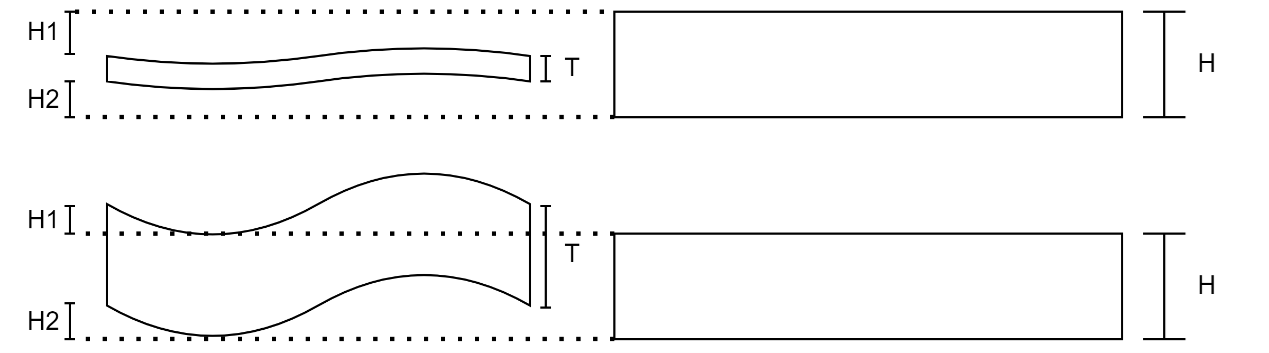

Supplement: Supplementary file 4 [file Image4.png]

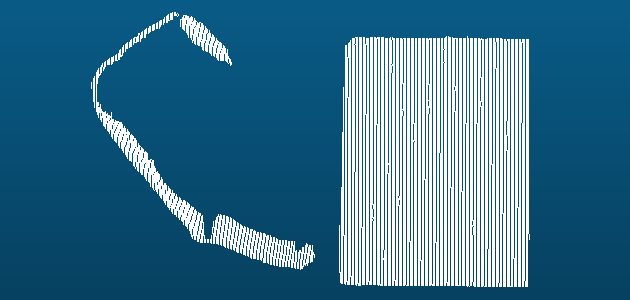

Supplement: Supplementary file 5 [file Image5.png]

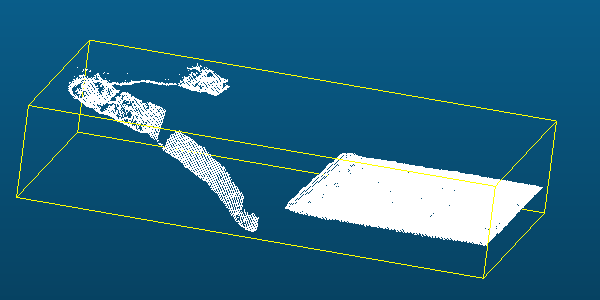

Supplement: Supplementary file 6 [file Image6.png]

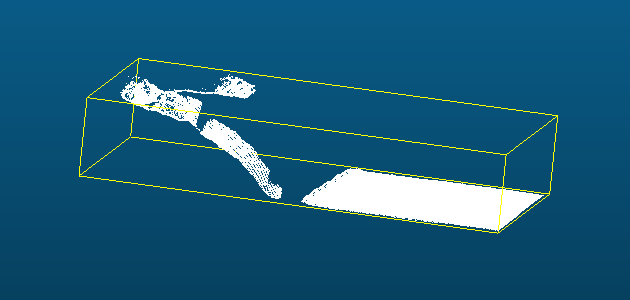

Supplement: Supplementary file 7 [file Image7.png]
