# Supplementary material for: Segmentation of tobacco shred point cloud and 3-D measurement based on improved PointNet++ network with DTC algorithm
Source: Front Plant Sci. 2025 Jan 21;15:1508449. doi: 10.3389/fpls.2024.1508449 (PMC11790634; doi:10.3389/fpls.2024.1508449)
Supplement: Supplementary file 8 [file Table1.docx]

**Supplementary TABLE 1 |** Error comparison between traditional 2D algorithms and our 3D measurement methods（Length）

| Measured Object | Manual  measurement |  | Traditional 2D algorithmic measurement | |  | Our 3D algorithmic measurement | |
| --- | --- | --- | --- | --- | --- | --- | --- |
|  | Mean |  | Mean absolute  error | Mean  relative  error |  | Mean absolute  error | Mean  relative  error |
| Standard blocks | 30.00mm |  | 0.22mm | 0.73% |  | 0.05 mm | 0.17% |
| Cut Stem | 11.50mm |  | 0.85mm | 7.96% |  | 0.49mm | 4.39% |
| Tobacco Silk | 47.12mm |  | 3.47 mm | 6.91% |  | 1.64 mm | 3.74% |
| Reconsitituted Tobacco Shred | 19.70mm |  | 1.24mm | 6.78% |  | 0.62mm | 3.12% |
| Expanded Tobacco Silk | 12.40mm |  | 0.85mm | 7.68% |  | 0.51mm | 4.85% |

**Supplementary TABLE 2 |** Error comparison between traditional 2D algorithms and our 3D measurement methods（Width）

| Measured Object | Manual  measurement |  | Traditional 2D algorithmic measurement | |  | Our 3D algorithmic measurement | |
| --- | --- | --- | --- | --- | --- | --- | --- |
|  | Mean |  | Mean absolute  error | Mean  relative  error |  | Mean absolute  error | Mean  relative  error |
| Standard blocks | 9.00mm |  | 0.22mm | 0.73% |  | 0.05 mm | 0.17% |
| Cut Stem | 5.71mm |  | 0.69mm | 12.79% |  | 0.21mm | 3.90% |
| Tobacco Silk | 1.71mm |  | 0.18mm | 15.28% |  | 0.07 mm | 6.77% |
| Reconsitituted Tobacco Shred | 1.01mm |  | 0.15mm | 14.37% |  | 0.06mm | 5.91% |
| Expanded Tobacco Silk | 3.30mm |  | 0.31mm | 12.89% |  | 0.18mm | 5.56% |

**Supplementary TABLE 3 |** Errors in 3D measurement methods (Thickness)

| Measured Object | Manual  measurement |  | 3D algorithmic measurement | |
| --- | --- | --- | --- | --- |
|  | Mean |  | Mean absolute  error | Mean  relative  error |
| Standard blocks | 0.500mm |  | 0.006 mm | 1.12% |
| Cut Stem | 0.403mm |  | 0.053mm | 13.57% |
| Tobacco Silk | 0.187mm |  | 0.036 mm | 19.94% |
| Reconsitituted Tobacco Shred | 0.186mm |  | 0.044mm | 24.23% |
| Expanded Tobacco Silk | 0.229mm |  | 0.051mm | 23.98% |
